# Knowledge, Attitude, and Behavior of Parents Regarding Early Childhood Caries Prevention of Preschool Children in Western Region of Saudi Arabia: A Cross-Sectional Study

**DOI:** 10.3390/dj10120218

**Published:** 2022-11-22

**Authors:** Afnan A. Nassar, Buthaina A. Fatani, Othoob T. Almobarak, Shahad I. Alotaibi, Reem A. Alhazmi, Abdullah A. Marghalani

**Affiliations:** 1 Department of Preventive Dentistry, Division of Public Health, Faculty of Dentistry, Umm Al-Qura University, Makkah 2424, Saudi Arabia; 2 Dental Intern, Faculty of Dentistry, Umm Al-Qura University, Makkah 2424, Saudi Arabia

**Keywords:** attitudes, childhood, dental caries, parental, prevention, knowledge

## Abstract

The knowledge and beliefs of parents have an impact on their children’s general and oral health. The objective of this study is to assess knowledge, attitude, and behavior of parents and identify possible associated factors of early childhood caries (ECC) in the western region of Saudi Arabia. Parents aged 18 and older were invited to complete a self-administered, validated questionnaire. Questions covered demographics, knowledge, attitude, and behavior regarding ECC etiology and prevention. Of the 644 parents who completed the questionnaire, 51.4% were not aware of the early signs of tooth decay, such as white lines. Pain and trauma were reported as the major reasons for a child to visit a dentist (42.4%). Only 37% of parents were aware of the importance of fissure sealant, and only 23% knew how it is applied. Compared with mothers, fathers were significantly less aware of hidden sugar and the relation between the frequency of consuming breast milk or bottled milk and caries development (*p* = 0.001). Similarly, fathers were more likely to believe that children could achieve effective teeth cleaning without parental assistance (*p* = 0.001). Preventive guidance should be provided by oral healthcare providers in Saudi Arabia to improve parents’ knowledge of ECC prevention.

## 1. Introduction

According to the American Academy of Pediatric Dentistry, early childhood caries (ECC) is defined as “the presence of one or more decayed (non-cavitated or cavitated lesions), missing (due to caries), or filled tooth surfaces in any primary tooth” in a child under the age of six [1]. ECC can begin early in a child’s life and advance quickly, particularly in children who are at high risk of developing dental caries. ECC may have significant consequences for children’s physical, psychological, and social well-being, since oral discomfort and tooth loss can have a negative impact on mastication and phonetics, which is reflected in the overall health-related quality of life (OHRQoL) of preschoolers as well as their families [2]. In addition, ECC in children at a very young age can be challenging, necessitating, in some cases, dental treatment under sedation or general anesthesia, which can be expensive and time-consuming and can lead to complications [3].

Bottle-feeding throughout the night, a poor diet high in sugar, lack of parental awareness of oral health, low socioeconomic status, and lack of access to dental care facilities have all been identified as risk factors for ECC [4,5]. Furthermore, visible plaque accumulation due to difficulty in performing proper oral hygine on teeth due to crowding, tooth morphology types, or malocclusion were also associated with dental caries [6]. Medical conditions or syndromes associated with enamel hypoplasia, physical impairment, decrease in the salivary flow, crowding due to supernumerary teeth, and the presence of cleft lip or palate were also reported as risk factors for ECC [5,7]. Examples of such syndromes are Sanjad–Sakati syndrome (Middle East syndrome), Down syndrome, and Orofaciodigital syndrome [7,8,9].

Early dental examination is essential in preventing ECC and in controlling an existing condition. It has been discovered that the knowledge and beliefs of parents have an impact on their children’s dental health [10]. Mothers’ oral health awareness and oral-health-related habits, such as tooth brushing, as well as their diet and food choices, were directly related to their children’s dental health [11]. Highly educated mothers showed a better understanding of oral hygiene and the importance of deciduous teeth compared with mothers with less education [11].

Dental caries is associated with multiple risk factors. It has been found that people’s behavior is shaped by social, economic, and environmental factors, which translate into positive health choices and practices [12].

Understanding the causes of ECC and oral hygiene practices in the Saudi population is crucial because a high prevalence of ECC (73%) was reported among pre-school children in the cities of Riyadh and Jeddah [13]. A study in China reported that severe ECC was found in 40.1% (*n* = 269) of children, with a mean DMFT (decayed, missed, and filled permanent teeth) of 7.72, with caries left untreated in more than 99% of cases. Higher ECC prevalence was shown to have statistically significant relationships with higher age and lower socioeconomic status [14]. Other studies have shown that caries in preschool children is linked to low parental knowledge, attitudes toward oral health, and lack of awareness about hidden sugars and their effects [15,16].

Only a few previous studies have investigated the awareness and knowledge of Saudi parents regarding ECC, and these studies had a limited sample size and were conducted at one location, making additional investigations necessary [17,18]. Assessing parents’ levels of knowledge on a wider scale can guide healthcare givers and dentists to improve parents’ knowledge and encourage them to advocate the use of available dental services to prevent ECC and avoid related complications.

The purpose of this study was to describe the knowledge, attitudes, and behaviors of parents regarding early childhood caries development and prevention in the western region of Saudi Arabia as well as to identify factors associated with ECC in the Saudi population, such as gender, level of education, family income, and the sources of parental knowledge. Only a few Saudi studies were found and none among the population of Makkah regarding parental knowledge and their effect to ECC formation and prevention.

## 2. Materials and Methods

This cross-sectional study assessed the knowledge, attitude, and behavior related to ECC prevention of parents of preschool children in the western region of Saudi Arabia. The study was approved by the ethical research committee at Umm Al-Qura University, Faculty of Dentistry, Makkah, Saudi Arabia (IRB number HAPO-02-K-012-2021-09-440). Using a sample size calculator with a confidence interval of 95%, a marginal error 5%, and a population proportion of 50%, it was determined that 385 participants needed to be included in this study. Taking into consideration possible selection bias and low responses from questionnaires, 800 parents were invited to participate in this study.

Convenience sampling was used to recruit male and female Arabic-speaking parents aged 18 years or older, who live in the western region of Saudi Arabia. The questionnaire was first developed in English and later translated into Arabic. Questionnaire validation was conducted via pilot testing of the survey on 20 participants of our intended population who were parents of our University Hospital pediatric patients. Then reliability testing was performed to check whether the responses were consistent (Cronbach’s Alpha score of 0.8 was found). The questionnaire was distributed as an on-line survey via social media channels to parents attending Umm Al-Qura University Dental Hospital. The participants provided their informed consent before completing the structured, self-administered questionnaire, which consisted of closed-ended questions. The time required to complete the questionnaire was approximately 10 min.

The questionnaire, which was adopted from previous studies [15,19,20], is composed of three sections. The first revolves around demographic characteristics, including age, gender, nationality, address, level of education, and parent occupation. The second and third sections comprise questions regarding parents’ knowledge levels and attitude regarding indicators of dental caries and ECC prevention.

Data were collected using Google Forms, tabulated, and statistically analyzed using Statistical Package for Social Science (SPSS v20) using descriptive statistics. The chi-square test was used with a significance level at (*p* < 0.05) to assess possible association between level of knowledge and demographic factors.

## 3. Results

Participant recruitment was conducted over the course of six months, with 800 prospective respondents invited to take part in the research. Of these, 644 completed the questionnaire, which corresponds to a response rate of 80.5%. The participants’ demographic data are summarized in Table 1. The majority were mothers, 560 (87%), and the remainder, 84 (13%), were fathers. More than half of the participants were aged 31–59 years old, 42 (66%), and were from Makkah city, 439 (68.2%). More than half of the participants have a college degree, 365 (56.7%). Fathers reported having various occupations, whereas most of the mothers were housewives, 475 (71%). A total of 278 (43.2%) respondents reported having a family income of SAR 7000 or less.

The results regarding parental knowledge about ECC are displayed in Table 2. The highest correct responses were associated with the following items: the time of eruption of first primary teeth and its importance, 441 (68.5%) and 465 (72.2%), respectively; basic preventive measures, such as controlling the diet of a pregnant mother, 451 (70%), and that of her child, 612 (95%), and the importance of controlling candy consumption by children and its helpfulness in the prevention of tooth decay, 623 (96.7%); the importance of brushing primary teeth and using fluoridated toothpaste, 637 (98.9%) and 477 (74.1%), respectively; and the use of dental floss whenever there is contact between teeth, 477 (74.1%).

Participants showed a gap in knowledge regarding early signs of caries, which are represented as white lines or spots, 331 (51.4%). Only 32% knew about bacterial transmission through item-sharing or kissing or its role in caries development. More than half of the respondents were unaware of professionally applied preventive measures, such as fissure sealants and their function and the techniques by which they are applied, 405 (62.9%) and 498 (77.4%), respectively. Nearly all the participants, 632 (98.1%), agreed that they need more information about ECC and that schools should focus on teaching oral health care. Many of the parents (46%) had misconceptions or did not know about bottle/breastfeeding and its link to ECC at night.

More than three-quarters of the parents believed that dental visits should be done every six months for early caries detection, 495 (76.9%), but this knowledge was not reflected in their behaviors because 42.4% of the parents took their children to visit a dentist only in case of pain or trauma. Parental knowledge about the types of cariogenic food and beverages was satisfactory, but the respondents did not know about the best time and frequency for consuming cariogenic food and beverages. The majority of the parents (81.4%) gave snacks to their children between meals. Most of them believed that maintaining the oral health of a child is a parent’s duty because the effective cleaning of teeth cannot be achieved by the child alone 630 (97.8%).

Table 3 shows participants’ behavior regarding their children oral health and ECC. Only half of the parents took their children to visit a dentist. They preferred dentists (58.2%) and social media (26.6%) as sources of information about oral health care. Gender played a role in the knowledge and behavior regarding ECC. Among the respondents, compared with the mothers, the fathers were significantly less aware of issues, such as hidden sugar in food and the association between the frequency of breastfeeding or bottle feeding with ECC, especially at night (*p* = 0.001). The fathers disregarded the importance of cleaning babies’ mouths even before teeth eruption (*p* = 0.03), and unlike the mothers, they believed that children can achieve effective teeth cleaning without parental assistance (*p* = 0.001). Low family income decreased the chances that a child will receive early treatment among families. Those with incomes of SAR 7000 or lower had not sought any form of dental treatment, 156 (56%), and did not believe that fillings for primary teeth are necessary, 146 (53%) (*p* = 0.001). More than half of the participants were unaware of pit and fissure sealants, especially those who depended on social media as their source of information (73.7%). This proportion was considered significant compared with those who depended on dentists as information sources (*p* = 0.003).

## 4. Discussion

Parental knowledge level and attitude are critical in preventing oral diseases and promoting children’s dental health. In this study, parents’ knowledge was generally acceptable, however, an obvious low level of knowledge was found with regard to causes and early signs of ECC, preventive measures, the importance of the first dental visit, and the best time for the consumption of sugary food and snacks.

The current study found a higher percentage of wrong answers associated with low-income parents compared with another study in Saudi Arabia [19]. Mothers were more knowledgeable than the fathers in many aspects in the present study, similar to the other Saudi study which found considerably higher knowledge among mothers (87%) compared with fathers (13%) [19]. The difference between parental knowledge levels could be due to the fact that mothers frequently accompany their children to dentist appointments and that they are more concerned about their children’s health.

Parental knowledge regarding the importance of fluoride for dental health was higher in the present study than in previous studies [19,21,22]. This could be due to dentifrice companies’ repeated advertisements which reinforced fluoride importance in their products. Fluoride has been proven to be effective in caries prevention; however, some studies argued that using fluoridated toothpaste twice daily among children younger than six years old has not been proven to inhibit ECC [23].

With regard to dental visits, 42.4% of parents in the current research took their children to the dentist only when their children were in pain or were suffering from trauma; this is consistent with other studies [18,19]. Regarding the appropriate time for a child’s first visit to the dentist, an extremely small percentage of our sample (5.7%) were aware that an infant requires a dental visit. Furthermore, only 12.4% of the participants were aware that the first dental visit should be scheduled within 6–12 months of tooth eruption. Similar percentages were reported by Alsane and his colleagues in Kuwait [12,18]. A slightly higher percentage (28%) was reported by a study conducted in Riyadh City, Saudi Arabia [13]. An emphasis on the importance of regular dental visits to help in early caries detection and prevention is crucial and early education directed towards pregnant mothers may be appropriate.

The majority of parents (81.4%) in our study thought that sugary and cariogenic food were better consumed between meals than during or at the end of meals. This finding is critical regarding the effects of the initiation and progression of caries. Sugar consumption was reported in many studies as a major risk factor among their population [24,25]. Chi and Scott [26] discovered strong links between parental sugar consumption and their children’s sugar consumption. Parents who frequently consume sugary foods and beverages may have a negative impact on their children’s diet. Furthermore, they discovered that children were naturally drawn to sweet food as opposed to vegetables and bitter foods [21,22,27]. Sugary foods and snacks were also sometimes used as a reward for positive behavior by parents, leading to overconsumption [26]. This finding showed that there is a need to focus on the parent’s behavior when designing an educational program for ECC prevention.

In our study, parents had a misconception about bottle/breast feeding frequency and its association with caries formation, reporting that they fed their children frequently, either by breast or bottle feeding, during the night (46%) and daytime (69.5%). A similar study in Abha City and Makkah City in Saudi Arabia found that more than half of the parents said they thought that nighttime bottle feeding would not affect their children’s primary dentition [3,10] compared with the population in the Kuwaiti study where only one-third of the study participants were properly informed about the risks of nighttime bottle or breastfeeding [18]. A study reported that breast fed children were significantly less likely to develop caries compared with those who were bottle fed from birth, while ECC was found among breast-fed children at night after the age of 6 months [28]. Although breastfeeding is the best nutritional source for newborns, mothers should be aware of its risk of devolving ECC and make sure to clean their child’s oral cavity after feeding.

Only 32% of our study’s sample knew about bacterial transmission by item-sharing or kissing and its role in caries development. These findings were similar to those of studies conducted in Brunei and Malaysia [15,29]. Targeting pregnant mothers could be an effective strategy to educate them about night-feeding side effects and about bacterial transmission, which can play a vital role in ECC prevention.

Even though all of the parents who took part in the Chiau study understood the importance of brushing their children’s teeth for excellent oral health, only 82.3% felt that brushing their children’s teeth as soon as they erupted was necessary [29]. Furthermore, just 45.9% of the participating parents reported brushing their children’s teeth on a regular basis, which indicates that knowledge alone does not always affect attitude and behavior. Parental behavior related to correct oral hygiene practices and training of their children accordingly is important since health habits are developed early in childhood and last into adulthood. Most parents (83.9%) in this study reported that their children brushed their teeth after every meal compared with other studies that reported that children brushed their teeth twice daily [19]. It may be the majority practice of this sample to brush after each meal—or it is what parents felt is better to report—either way, parents should encourage their children to maintain at least twice a day brushing habits and not to ignore the one before bed time. In our study, 63% of the parents said they did not think that effective cleaning could be achieved by the child alone when the child is too young to exert his/her own independence. In contrast, in a Malaysian study, 52% of parents thought that the effective cleaning of teeth could be achieved by the child alone [15]. Dentists can provide appropriate training for children, those with proper age and skills, in front of their parents during dental visits. This will ensure that adult supervision and monitoring of their children’s oral hygiene skills are provided.

Nearly half of this study’s participants recognized white spots on the teeth as an early sign of tooth decay. In contrast, another study showed that only 8% of the parents recognized the significance of white spots [30]. In this study, 72.2% of the parents said they were aware of the importance of primary teeth, with nearly half of them understanding that primary teeth could be filled if needed. These results are fairly similar to those of a study that reported that approximately 69% of parents indicated that primary teeth are very important and should be filled when necessary [30].

This study also found that 58.2% of the parents preferred the dentist as their source of dental information, while 26% preferred social media as their main source in obtaining knowledge about oral health. A cross-sectional study showed that 94% of the participating parents reported that the dentist was their most important source for oral health information [19]. Others reported that gynecologists and pediatricians were the participants’ most relied on sources of ECC knowledge [18]. Pediatricians and family physicians are more frequently seen by children in their initial years of life. It is important to acknowledge pediatrician and family physician roles in the promotion of children’s oral health and how they can advise families to start dental visits [13]. Since many participants depended on social media as their knowledge source, oral health educational campaigns should target social media audiences using their preferred platforms and stream the correct and understandable dental educational material. Today, social media platforms play a major role in our lives in entertainment as well as in education. Millions of social media followers use different platforms to search for information, which makes it easy to target these audiences and deliver planned educational messages. Some studies evaluated educational videos posted on YouTube and reported that mouth sores information was slightly useful in 68.97% of the analyzed videos [31]. In addition, evaluation of the usefulness of the posted content can be easily tracked. A study found that the success and usefulness of social media videos on children’s oral thrush were associated with the number of viewings, number of likes, and viewing rate [32].

A limited number of fathers participated in this survey, which is considered a limitation of this study and may affect generalizability of the results. Additionally, the study’s largest number of participants were from Makkah City, with fewer participants from other cities and towns in the western region. Future studies should aim to recruit more fathers and include more participants from different geographical areas. Using an online-based survey may not be representative of all parents in the western region; a phone-call-based survey might provide a wider range of data from different socioeconomic areas.

This study’s findings can help fill the gap in knowledge and provide an update, which can guide the development of community services and caries prevention programs for children and their caregivers run by oral healthcare providers. The development of a well-planned ECC prevention program targeting parents accompanied with their children should be accomplished via collaboration with the Ministry of Health and the Ministry of Education. It is important for dentists and dental care-givers to follow trends in the society and to use social media platforms as a planned source of education to target large audiences.

## 5. Conclusions

Parental knowledge and behavior regarding caries formation and prevention were found to be not satisfactory. Fathers showed less awareness compared with mothers in multiple aspects. Prophylactic guidance should be reinforced by healthcare and oral healthcare providers to improve parental knowledge in Saudi Arabia.

## Figures and Tables

**Table 1:** Participants’ demographic data.

Questions	Answers	Frequency (%)
1—Gender	(A) Male (B) Female	84 (13%) 560 (87%)
2—Age	(A) 18–30 Y (B) 31–59 Y (C) 60 Y and more	206 (32%) 429 (66.6%) 9 (1.4%)
3—Nationality	(A) Saudi (B) Non-Saudi	545 (84.6%) 99 (15.4%)
4—City/Home address	(A) Makkah (B) Jeddah (C) Madina (D) Taif (E) Other	439 (68.2%) 74 (11.5) 18 (2.8%) 45 (7%) 68 (10.6%)
5—Educational level	(A) High school or less (B) College (C) Post graduate (D) Other	226 (35.1%) 365 (56.7%) 29 (4.5%) 24 (3.7%)
6—Father occupation	(A) Governmental (B) Private sector (C) Teacher/academic (D) Military (E) Medical field (F) Dentist (G) Retired (H) Not working (I) Other	196 (30.4%) 123 (19.1%) 45 (7%) 91 (14.1%) 27 (4.2%) 2 (0.3%) 76 (11.8) 61 (9.5%) 23 (3.6%)
7—Mother occupation	(J) Governmental (K) Private sector (L) Teacher/academic (M) Military (N) Medical field (O) Dentist (P) Retired (Q) Housewife (R) Other	59 (9.2%) 27 (4.2%) 59 (9.2%) - 10 (1.6%) 3 (0.5%) 12 (1.9%) 475 (71%) 17 (2.6%)
8—Family income/ month	(A) 7000 or less SAR (B) 8000–10,000 SAR (C) 11,000–16,000 SAR (D) More than 16,000 SAR	278 (43.2%) 142 (22%) 140 (21.7%) 84 (13%)
9—Number of children	(A) 1–2 children (B) 3–4 children (C) 5 or more children	253 (39.3%) 246 (38.2%) 145 (22.5%)

(*n* = 644), SAR = Saudi Arabian Riyals, Y = years.

**Table 2:** Parental knowledge regarding early childhood caries.

Questions	Answers	Prevalence
1—When does the first baby tooth appear in the child’s mouth?	(A) 4 months (B) 6 months (C) 12 months	115 (17.9%) 441 (68.5%) 88 (13.7%)
2—Do you think primary teeth are as important as permanent teeth?	(A) primary teeth are not important (B) slightly important (C) equally important	35 (5.4%) 144 (22.4%) 465 (72.2%)
3—Is it necessary to do filling in baby teeth when required?	(A) yes (B) no (C) I don’t know	362 (56.2%) 149 (23.1%) 133 (20.7%)
4—Do you think that you should visit the dentist once every 6 months?	(A) yes (B) no (C) I don’t know	495 (76.9%) 84 (13%) 65 (10.1%)
5—Do you think brushing your baby’s teeth is important?	(A) yes (B) no (C) I don’t know	637 (98.9%) 4 (0.6%) 3 (0.5%)
6—Do you think cleaning your baby’s mouth should begin even before teeth erupt?	(A) yes (B) no (C) I don’t know	385 (59.8%) 163 (25.3%) 96 (14.9%)
7—Do you think that tooth decay can affect infants below 2 years of age?	(A) yes (B) no (C) I don’t know	492 (76.4%) 59 (9.2%) 93 (14.4%)
8—Do you think white lines or spots on the tooth surfaces are the first signs of tooth decay?	(A) yes (B) no (C) I don’t know	313 (48.6%) 91 (14.1%) 240 (37.3%)
9—Do you think that tooth decay is caused by bacteria that are transmitted by sharing feeding utensils (e.g., spoon)	(A) yes (B) no (C) I don’t know	206 (32%) 250 (38.8%) 188 (29.2%)
10—Do you think that controlling candies/sweets of children will be helpful in the prevention of tooth decay?	(A) yes (B) no (C) I don’t know	623 (96.7%) 12 (1.9%) 9 (1.4%)
11—Do you think fluoride in toothpaste is important for preventing tooth decay for children?	(A) yes (B) no (C) I don’t know	477 (74.1%) 53 (8.2%) 114 (17.7%)
12—Do you know what pit and fissure sealants are?	(A) yes (B) no (C) I don’t know	239 (37.1%) 260 (40.4%) 145 (22.5%)
13—Pit and fissure sealants are applied to…	(A) sound teeth (B) carious teeth (C) I don’t know	146 (22.7%) 307 (47.7%) 191 (29.7%)
14—Does mother’s diet during pregnancy affect development of baby’s teeth?	(A) yes (B) no (C) I don’t know	451 (70%) 64 (9.9%) 129 (20%)
15—Do you think dental health education for children should be taught at school?	(A) yes (B) no (C) I don’t know	632 (98.1%) 4 (0.6%) 8 (1.2%)
16—Do you think balanced diet is essential for the healthy growth of a baby’s teeth?	(A) yes (B) no (C) I don’t know	612 (95%) 8 (1.2%) 24 (3.7%)
17—Do you think you need more knowledge regarding ECC prevention?	(A) yes (B) no (C) I don’t know	554 (86%) 74 (11.5%) 16 (2.5%)
18—Do you think effective cleaning of a child’s teeth can be achieved by the child him/herself?	(A) yes (B) no (C) I don’t know	226 (35.1%) 407 (63.2%) 11 (1.7%)
19—Do you think maintaining oral health of a child is the parent’s duty?	(A) yes (B) no	630 (97.8%) 14 (2.2%)
20—Do you think swallowing fluoride toothpaste can be harmful to a child’s health?	(A) yes (B) no (C) I don’t know	131 (20.3%) 314 (48.8%) 199 (30.9%)
21—When should you use dental floss for your child?	(A) 3 years (B) 6 years (C) when there is contact between the teeth	46 (7.1%) 121 (18.8%) 477 (74.1%)
22—Do you think that night time bottle and breast feeding can cause tooth decay?	(A) yes (B) no (C) I don’t know	342 (53.1%) 150 (23.3%) 152 (23.6%)
23—Frequent and prolonged breast/bottle feeding in the daytime can cause tooth decay?	(A) yes (B) no (C) I don’t know	197 (30.6%) 247 (38.4%) 200 (31.1%)

**Table 3:** Participants’ behavior regarding oral health and ECC.

Questions	Answers	Prevalence
1—Has your child received any dental treatment before?	(A) yes (B) no	362 (56.2) 282 (43.8%)
2—From where do you obtain the knowledge about oral healthcare?	(A) dentist (B) general practitioner (C) social media (D) TV (E) educational books (F) friends	375 (58.2%) 17 (2.6%) 171 (26.6%) 28 (4.3%) 30 (4.7%) 23 (3.6%)
3—When do you take your child to visit a dentist?	(A) when the first tooth appears (B) 1–3 years (C) 4–6 years (D) only in pain or trauma	37 (5.7%) 161 (25%) 173 (26.9%) 273 (42.4%)
4—When do you give sugary and cariogenic food (candies and sweet beverages) to your child?	(A) with meals (B) between meals (C) before going to bed	85 (13.2%) 524 (81.4%) 35 (5.4%)
5—Do you feed your child with items that you have used yourself for eating or drinking?	(A) yes (B) no	224 (34.8%) 420 (65.2%)
6—Has child used a sweetened baby bottle or honey-dipped pacifier?	(A) yes (B) no	98 (15.2%) 546 (84.8%)
7—Should the baby drink milk with a cup when he/she gets old enough to hold it?	(A) yes (B) no (C) I don’t know	599 (93%) 23 (3.6%) 22 (3.4%)
8—A child’s teeth should be cleaned/brushed as soon as the teeth erupt?	(A) yes (B) no (C) I don’t know	497 (77.2%) 101 (15.7%) 46 (7.1%)
9—Is it advisable to brush the teeth after every meal?	(A) yes (B) no (C) I don’t know	540 (83.9%) 81 (12.6%) 23 (3.6%)

## Data Availability

The datasets are available from the corresponding author on reasonable request.

## Abbreviations

ECC	early childhood caries
AAPD	American Academy of Pediatric Dentistry
DMFT	decayed, missed, and filled permanent teeth
OHRQoL	ral-health-related quality of life
IRB	Institutional Review Board
SPSS	Statistical Package for Social Science
SAR	Saudi Arabian Riyals
Y	years
